# Protective effects of selenized yeast on the combination of cadmium-, lead-, mercury-, and chromium-induced toxicity in laying hens

**DOI:** 10.3389/fvets.2022.958056

**Published:** 2022-09-29

**Authors:** Caimei Wu, Jingping Song, Lang Li, Yuxuan Jiang, Todd J. Applegate, Bing Wu, Guangmang Liu, Jianping Wang, Yan Lin, Keying Zhang, Hua Li, Fali Wu, Shiping Bai

**Affiliations:** ^1^Key Laboratory for Animal Disease-Resistance Nutrition and Feedstuffs of China Ministry of Agriculture and Rural Affairs, Institute of Animal Nutrition, Sichuan Agricultural University, Chengdu, China; ^2^Department of Poultry Science, University of Georgia, Athens, GA, United States; ^3^Chelota Biotechnology Co., Ltd., Deyang, China

**Keywords:** heavy metal, chromium, selenium, intoxication, laying hens

## Abstract

The objective of this study was to investigate the toxic effects of a combination of cadmium (Cd), lead (Pb), mercury (Hg), and chromium (Cr) on laying performance, egg quality, serum biochemical parameters, and oxidative stress of laying hens, as well as the alleviating action of dietary supplementation of selenized yeast. A total of 160 Lohmann pink-shell laying hens (63-week-old) were randomly divided into four treatments with 10 replicates of four hens each. The treatments were the corn–soybean meal basal diet (control; CON), the CON diet supplemented with 0.4 mg selenium (Se)/kg from selenized yeast (Se); combined heavy metals group: the basal diet supplemented with 5 mg Cd/kg, 50 mg Pb/kg, 3 mg Hg/kg, and 5 mg Cr/kg (HEM), and the HEM diet supplemented with 0.4 mg Se/kg from selenized yeast (HEM+Se). The experimental period lasted for 12 weeks. The HEM diet decreased hen-day egg production, feed conversion ratio (FCR), and egg white quality (*P* < 0.05), but increased (*P* < 0.05) glutamic oxalacetic transaminase (AST) activity in the serum. HEM induced higher malondialdehyde (MDA) and reactive oxygen species (ROS) in the serum, liver, and ovary and significantly decreased (*P* < 0.05) the activity of total superoxide dismutase (SOD) and tended to decrease glutathione S-transferase (GST) (*P* = 0.09) in the serum. Meanwhile, HEM significantly decreased (*P* < 0.05) activity of SOD, GST, glutathione peroxidase (GPX), and glutathione (GSH) in the liver, and the activity of GPX and GSH in the ovary. Se addition of 0.4 mg/kg significantly (*P* < 0.05) improved hen-day egg production and FCR and decreased AST concentration and increased some enzyme activity in the serum, liver, and ovary. In conclusion, dietary HEM exposure depressed laying performance, and egg white quality was likely due to an impaired antioxidant capacity, disrupted hepatic function, and elevated HEM accumulation in the egg yolk and egg white of laying hens. Se addition of 0.4 mg/kg ameliorated toxic effects of HEM on laying performance, oxidative stress, and hepatic function.

## Highlights

- Cadmium, lead, mercury, and chromium are common environmental pollutants, which often occur together contaminating the feedstuff.
- The study aimed to investigate the toxic effects of a combination of cadmium, lead, mercury, and chromium on laying performance, egg quality, serum biochemical parameters, and oxidative stress of laying hens, and the alleviating action of selenized yeast on these effects.
- Our findings will provide a valuable insight into the toxicity of combined heavy metals on laying hens, as well as ameliorative effects of selenized yeast.

## Introduction

High cadmium (Cd), lead (Pb), and/or mercury (Hg) contamination are toxic to the poultry health. Air, sewage, soil, manure, forage crops, processing plants and mineral supplements, and premixes are the primary heavy metal contamination sources (1). Mineral supplements and premixes generally contain higher Cd and Pb concentrations than the other main components of the ration (2). Protein hydrolysates from poultry (chicken and turkey) feathers as feather meal or fish meal are considered as a main source of Hg contamination in animal feed (3). Cd, Pb, and Hg accumulate in the body tissues, primarily the kidneys and liver (4–7), and cause toxicity. It has been reported that Cd (8–11), Pb (12–15), and Hg (16, 17) have negative effects on laying performance and egg quality, oxidative stress, disease, histopathological damage, and hormone of female poultry.

Chromium (Cr) is an essential mineral in livestock animals. Almost all naturally found Cr is trivalent, while hexavalent Cr is mostly of industrial origin. Trivalent Cr tends to accumulate in epidermal tissues and in bones, liver, kidney, spleen, lungs, and the large intestine (18). The toxicity of trivalent Cr is low (19). Some previous studies have shown that organic (chromium yeast, chromium nicotinate, or chromium picolinate) or inorganic Cr [chromium chloride (CrCl_3_)] had no effect or a slight increase in egg production (20–22). However, some studies indicated that trivalent Cr as different chemical forms had an adverse effect on laying hens. Mariottini et al. (23) found that high concentrations (50 mg/kg) and different chemical forms (chromium yeast and chromium aminoniacinate) of trivalent Cr supplementation can substantially impair hepatic metabolizing cytochrome P-450 (CYP)-linked enzymes in laying hens. Cr as chromium propionate at 400 μg/kg decreased albumen height, yolk color score, and Haugh unit of eggs (24). Dietary supplementation with chromium picolinate decreased serum glucose concentration of Beijing Red brown-egg laying hens (25).

While in the actual commercial laying hen production, co-contamination with many kinds of heavy metals exists simultaneously. Some reports have shown that the combined feeding of Pb and Cd leads to liver oxidative damage of laying hens (26) and decreased laying performance and egg quality (27). Kim et al. (28) found that in-feed heavy metals for layer diets up to 30 mg Pb/kg and 1.2 mg Hg/kg decreased F_1_ follicle weights simultaneously causing hepatic dysfunction as indicated by increasing blood metabolites that are associated with liver inflammation. Meanwhile, combined administration of methylmercury chloride (MeHgCl), lead acetate (PbAc), and cadmium chloride (CdCl_2_) increased the severity of hepatic histopathologic changes and disturbed hepatic metal concentrations in Pekin ducks (29, 30).

Selenium is an essential micronutrient with antioxidant function for animals and can alleviate heavy metal-induced poisoning (31). Some studies conducted with laying hens have revealed the ameliorative effects of Se against Cd-induced kidney and ovarian damage (32, 33). Se alleviates Pb-induced oxidative stress and immune damage in the bursa of Fabricius of chicken (34) and immune toxicity in the hearts of chickens. Caban et al. (35) observed that an antagonistic effect of Se on Hg toxicity involves the liver of female broiler chickens. In addition, diphenyl diselenide [(PhSe)_2_] decreases methylmercury-induced cerebral, hepatic, and renal oxidative stress and Hg deposition in adult mice (36). It has been reported that Se administration alleviates Cr toxicity in the chicken brain and liver (37, 38).

Cadmium, lead, mercury, and chromium are frequently found together in the aquatic environments and can occur in high concentrations in animals, feedstuffs, and edible tissues (39–41). To date, there is little available information on the effects of combined administration of Cd, Pb, Hg, and Cr on laying performance, egg quality, and oxidative stress of laying hens, and whether these detrimental effects could be alleviated with Se. Meanwhile, the maximum permitted limits of Cd, Pb, Hg, Cr, and Se in feedstuff are 0.5, 5, 0.1, 5, and 0.5 mg/kg in China, respectively (42), but due to the loosely controlled quality of raw materials, the contents of Cd, Pb, Hg, and Cr especially Pb and Cr in formula feed may exceed the limits of national standards. The maximum tolerable levels of Cd, Pb, Hg, and Cr for poultry are 0.5, 30, 2, and 1,000 mg/kg, respectively (43), but to construct toxicity models of chronic or sub-chronic, the concentration of Cd, Pb, or Hg in the poultry feed was used up to 420-, 200-, and 270-fold of the maximum permitted concentration in several studies, and the test period was 8, 10, or 12 weeks (10, 12, 17). Therefore, to construct the laying hen model of sub-chronic heavy metal intoxication, the addition of 5 mg/kg Cd, 50 mg/kg Pb, 3 mg/kg Hg, and 5 mg/kg Cr approximates the 10-fold of the limits of national standards, were chosen in this study. In addition, our previous research has indicated that the HEM dosages have disturbed the ion balance of reproductive organs of laying hens (44). Meanwhile, the maximum permitted limits of Se in poultry feed are 0.5 mg/kg, based on the Se content 1.4 mg/kg in basal dietary, 0.4 mg/kg Se from selenized yeast was used in this study.

Thus, the objective of this study was to investigate the combined effects of dietary Cd, Pb, Hg, and Cr on laying performance, egg quality, serum biochemical and hormone indicators, and oxidative stress of laying hens, and whether these effects could be attenuated with selenized yeast supplementation to the diet.

## Materials and methods

### Materials

Cadmium chloride (CdCl_2_), lead nitrate [Pb (NO_3_)_2_], mercury chloride (HgCl_2_), chromium chloride (CrCl_3_), nitric acid (HNO_3_), perchloric acid (HClO_4_), and hydrogen fluoride (HF) were purchased from Kelong company of Chengdu (Sichuan, China). Selenized yeast which contains 0.2% Se was provided by Chelota biotechnology Co., Ltd (Deyang, Sichuan, China). Lohmann pink-shell laying hens were obtained from commercial layer farms (Mianyang, Sichuan, China).

### Experimental design and management

One hundred and sixty (63-week-old) Lohmann pink-shell laying hens were randomly divided into four treatments with 10 replicates of four hens each. The four treatments included the corn–soybean meal basal diet (control; CON), the CON diet supplemented with 0.4 mg Se/kg from selenized yeast (Se), the CON diet supplemented with 5 mg Cd/kg from CdCl_2_, 50 mg Pb/kg from Pb(NO_3_)_2_, 3 mg Hg/kg from HgCl_2_, and 5 mg Cr/kg from CrCl_3_ (HEM), and the HEM diet supplemented with 0.4 mg Se/kg from selenized yeast (HEM+Se). The corn–soybean meal basal diet (Table 1) was formulated to meet the nutrient requirements of laying hens recommended by the National Research Council (45). All birds were reared in an environmentally controlled house. The temperature and humidity were set at 25°C and 65%, respectively. There were two birds in one cage (L × W × H = 38.5 × 38 × 34 cm), and two adjacent cages belonged to one replicate unit. A photoperiod of 16-h light and 8-h dark was maintained. The experiment lasted 12 weeks, and water and feed were provided *ad libitum* during the experimental period.

**Table 1:** Composition and nutrient concentrations of the basal diet (air-dry basis, %).

**Ingredients**	**Amount (%)**	**Calculated nutrients levels**	**Amount (%)**
Corn	58.11	ME (MJ/kg)	10.90
Soybean meal	16.60	Crude protein	14.56
Wheat bran	6.48	Calcium	3.55
Rapeseed meal	2.80	Non-phytate phosphorus	0.33
Corn distillers dried grains with soluble	3.00	Lysine	0.75
Soybean oil	1.80	Methionine	0.33
CaCO_3_	8.40	Methionine + cysteine	0.60
CaHPO_3_·H_2_O	1.20	Threonine	0.57
L-Lysine hydrochloride	0.18	Tryptophan	0.18
NaCl	0.40		
Choline chloride	0.10		
Vitamin premix^a^	0.03		
Mineral premix^b^	0.90		

a Vitamin premix provided the following per kilogram of complete diet: vitamin A (retinyl palmitate) 10,000 IU, vitamin D 2,500 IU, vitamin E (DL-tocopheryl acetate) 6.25 IU, vitamin k_3_ 1.25 IU, thiamine 0.5 mg, riboflavin 4 mg, pantothenic 6.25 mg, niacin 8.75 mg, pyridoxine 1.5 mg, biotin 0.0125 mg, folic acid 0.125 mg, VB12 0.0075 mg.

b Mineral premix supplied the following per kilogram of complete diet: Fe (FeSO_4_·H_2_O),60 mg; Cu(CuSO_4_·5H_2_O), 8 mg; Zn(ZnSO_4_·H_2_O), 80 mg; Mn(MnSO_4_·H_2_O), 60 mg; I(KI) 0.35 mg.

### Laying performance

The numbers of total eggs produced, salable eggs, soft shell eggs, broken eggs, dirty eggs, and misshapen eggs were recorded every day. Feed consumption of each replicate was recorded weekly. Hen-day egg production, average daily feed intake, and average egg weight were calculated. The feed conversion ratio was calculated as feed consumption divided by the total egg weight (feed/egg, g/g).

### Samples collection

After the experiment, 30 eggs from each treatment (three eggs per replicate) were collected to determine egg quality parameters. The egg yolk and egg white of every three eggs from each replicate were pooled to one sample for the mineral analyses. Two birds were randomly selected from each replicate. Ten ml of blood per bird was collected from the right-wing vein and then was centrifuged at 2,000 × g for 10 min at 4°C to collect the serum sample. After the blood collection, all birds were euthanized by cervical dislocation, and the left liver and the ovary tissue without the follicles were collected and stored in liquid nitrogen for antioxidant indicators analyses.

Samples of 0.1–0.2 g of liver or ovaries were homogenized in 9 ml of 0.9% sodium chloride buffer on ice using the Ultra-Turrax homogenizer (Tekmar Co., Cincinnati, OH) for 10 s and centrifuged at 1,500 × g for 15 min at 4°C, and the supernatant was used for the following analysis of antioxidant indicators.

#### Egg quality

The egg weight, yolk weight, yolk color, Haugh unit (HU), albumin height, eggshell strength, eggshell thickness, and eggshell color were determined in the fresh eggs (9). The egg weight and yolk weight were individually weighed. The yolk weight percent was calculated as presented by the percentage of the whole egg weight. The yolk color, HU, and albumin height were measured using an Egg Multi Tester (EMT-7300, Robotmation, Tokyo, Japan). The eggshell strength was measured by an eggshell force gauge (model II, Robotmation, Tokyo, Japan). The eggshell thickness was determined by an eggshell thickness gauge (ETG-1601A, Robotmation, Tokyo, Japan) at the large end, equatorial region, and small end of the egg. Eggshell color was measured by colorimeter (CR-400, Shanghai, China).

#### Minerals analyses

The samples of egg yolk and white were lyophilized using vacuum freeze dryer (FDU-2110, EYELA, Tokyo, Japan) prior to minerals analyses. Approximately 0.5 g of lyophilized egg yolk or white sample was transferred into a polytetrafluoroethylene digestion vessel. The 10 ml of concentrated HNO_3_ and HF (HNO_3_: HF = 4:1, v/v) was added to each vessel, and then the vessels were closed and placed into the microwave digestion system (MARS6, CEM Corporation, Matthews, NC, USA). After evaporating the digestion liquids to near dryness using a heater (BHW-09Y, Botonyc, Shanghai, China), the residuals were re-dissolved with 0.5% HNO_3_ in a volumetric flask. The concentrations of Pb, Cd, and Cr in the final solutions were measured by a graphite furnace atomic absorption spectrometer (Contr AA700, Analytik Jena AG, Jena, Germany). The concentration of Hg was determined using a Hg determination instrument (DMA-80, Evo, Milestone, Italy). For the Se examination, approximately 1.0 g of lyophilized egg yolk or white sample was transferred into a conical flask and then digested with by 10 ml of acid mixture (HNO_3_ to HClO_4_ = 4:1, v/v). The digestion was kept at 180°C using an electric sand bath (DK-2, Tiantan Instrument Co., Ltd, Tianjin, China) until the liquid in the conical flask became clear and colorless. The liquid remaining in the flask was transferred to a 50-ml volumetric flask, and then 2 ml of 200 g/L potassium ferricyanide solution and 16% HCl solution of HCl were added into the volumetric flask. Se concentration was measured using an atomic fluorescence spectrometer (AFS-230E, Haiguang Instrument, Beijing, China).

The limits of detection of these methods were Cd, 0.01 mg/kg; Cr, 0.02 mg/kg; Pb, 0.01 mg/kg; Hg, 0.0005 mg/kg; and Se, 0.00031 mg/kg, respectively. A standard reference of wheat powder provided by National Institute of Standards and Technology (Beijing, China) was included in each batch of analysis to verify the determination validation. The R^2^ values of standard curves for the different heavy metals were higher than 0.99. The recovery rates of Cd, Pb, Cr, Hg, and Se were 91.8–98.4%, 93.9–97.4%, 96.9–100.3%, 97.9–103.7%, and 92.9–102.1%, respectively (44).

### Serum biochemical parameters assays

The serum concentrations of total triglycerides (TG), total cholesterol (TC), high-density lipoprotein cholesterol (HDL-C), low-density lipoprotein cholesterol (LDL-C), glucose (GLU), total protein (TP), albumin (ALB), glutamic-pyruvic transaminase (ALT), glutamic oxalacetic transaminase (AST), and urea nitrogen (BUN) were determined using an automatic biochemical analyzer (Hitachi 3100, Tokyo, Japan) and the corresponding commercial kit (Maccura Biotechnology Co. Ltd., Chengdu, China). The serum concentrations of progesterone 4 (P4) and estradiol 2 (E2) were measured using a fluorescence microplate reader (SpectraMax M2, Molecular Devices, Sunnyvale, CA, USA) and the corresponding enzyme-linked immunosorbent assay (ELISA) kit (Shanghai Meilian Bioengineering Institute, Shanghai, China).

### Antioxidative parameters assays

In the samples of serum, liver, and ovary, the malondialdehyde (MDA) concentration, and the activities of total superoxide dismutase (T-SOD), glutathione (GSH), glutathione peroxidase (GSH-Px), and glutathione S-transferase (GSTs) were measured using the corresponding ELISA commercial kit (No. A006-1-1 for GSH, No. A004 for GST, No. A005 for GSH-PX, No. A001-3 for SOD, and No. A003-1 for MDA; Nanjing Jiancheng Bioengineering Institute, Nanjing, China) according to the manufacturer's instruction. The concentration of reactive oxygen species (ROS) was also determined using the ELISA kit (Shanghai Meilian Bioengineering Institute, Shanghai, China). Triplicate analyses were performed for each sample.

### Statistical analysis

Normality tests were performed for all data using the UNIVARIATE procedure of SAS (version 9.4, SAS Inst. Inc., Cary, NC, USA) with Normal and PLOT options, and then, Levene's tests were used to evaluate the heterogeneity of variances of data. The PROC MIXED procedure of SAS (version 9.4, SAS Inst. Inc., Cary, NC, USA) was used to analyze the data related to the laying performance, egg quality, serum biochemical parameters, and antioxidant parameters. The model used for analyzing laying performance was as follows: *Y*_*ijk*_ = μ+*T*_*i*_+*P*_*j*_+(*TP*)_*ij*_+*e*_*ijk*_, and the model used for analyzing the data of egg quality, serum biochemical parameters, and antioxidant parameters was as follows: *Y*_*ij*_ = μ+*T*_*i*_+*e*_*ij*_; where Y was an observation of the dependent variable, μ was the population mean for the variable, T_i_ was the fixed effect of treatment (CON, Se, HEM, or HEM+Se), P_j_ was the period effect, the TP was the interaction between dietary treatment and period, and e was the random error associated with the observation. The Tukey test was used for multiple population comparisons among different treatment means. The replicate was used as the experimental unit. If only there were two groups, the t-test procedure was used to compare the means between them. The relationships between different mineral concentrations and egg quality parameters were analyzed by the PROC CORR procedure of SAS, and the Pearson correlation option was used in our study. The results were reported as the mean and standard error of the mean (SEM). Values of *P* < 0.05 were considered statistically significant, and the tendency was declared at 0.05 < *P* < 0.10.

## Results

### Laying performance

Dietary HEM exposure significantly decreased (*P* < 0.05) average hen-day egg production and feed conversion ratio (FCR) as compared to the CON (Table 2) in the whole experimental period (weeks 1–12). Se supplementation showed the ameliorated effect on the HEM-induced laying performance toxicity (*P* < 0.05). Dietary HEM exposure did not influence (*P* > 0.10) average daily feed intake, average egg weight, broken eggs, salable eggs, soft shell eggs, dirty eggs, and misshapen eggs in weeks 1–12 compared to the CON. Moreover, with the time of dietary HEM exposure increasing, average hen-day egg production and FCR reduced in weeks 9–12 than those in weeks 1–4 and 5–8. However, other laying performance index did not change with the time of dietary HEM exposure (Table 2).

**Table 2:** Combined effects of dietary cadmium, lead, mercury, and chromium on laying performance of laying hens from 63 to 74 weeks of age and attenuated toxicity with selenized yeast.

**Items**	**Period**	**Main effect of diet**					**Main effect of period**				***P*** **-value**		
		**CON**	**Se**	**HEM**	**HEM+Se**	**SEM**	**week1-4**	**week5-8**	**week9-12**	**SEM**	**Diet**	**Period**	Diet^*^Period
Feed intake	week1-4	112.56	112.63	112.53	112.04								
	week5-8	112.54	111.77	112.72	111.81								
	week9-12	112.64	112.56	112.62	113.06								
	Overall	112.58	112.32	112.63	112.30	0.30	112.44	112.21	112.72	0.290	0.689	0.370	0.731
Feed conversion ratio^1^, g:g	week1-4	1.75	1.74	1.75	1.76								
	week5-8	2.14^c^	2.16^c^	2.15^c^	2.14^c^								
	week9-12	2.13^c^	2.13^c^	2.28^b^	2.14^c^								
	Overall	2.14	2.15	2.31	2.19	0.010	2.14^a^	2.17^a^	2.28^b^	0.010	<0.001	<0.001	<0.001
Hen-day egg production, %	week1-4	84.84^a^	84.07^a^	84.45^a^	84.67^a^								
	week5-8	84.70^a^	84.34^a^	79.35^b^	83.99^a^								
	week9-12	84.48^a^	83.93^a^	71.86^c^	79.30^b^								
	Overall	84.67	84.12	78.55	82.65	0.140	84.51^a^	83.10^a^	79.89^b^	0.140	<0.001	<0.001	<0.001
Average egg weight, g	week1-4	62.15	62.10	62.10	62.23								
	week5-8	62.40	62.30	62.30	62.13								
	week9-12	62.04	62.17	62.31	62.31								
	Overall	62.19	62.19	62.23	62.23	0.09	62.14	62.28	62.21	0.070	0.984	0.392	0.504
Egg disqualification ratio^2^, %	week1-4	1.08	1.08	2.26	2.40								
	week5-8	0.48	1.08	1.10	0.57								
	week9-12	2.26	0.48	2.28	2.06								
	Overall	1.69	1.07	1.55	1.04	0.460	1.52	1.37	1.08	0.360	0.400	0.153	0.131

CON = the corn–soybean meal basal diet; Se = the CON diet supplemented with 0.4 mg selenium/kg from selenized yeast; HEM = the CON diet added with 5 mg cadmium/kg from CdCl_2_, 50 mg lead/kg from Pb(NO_3_)_2_, 3 mg mercury/kg from HgCl_2_, and 5 mg chromium/kg from CrCl_3_; HEM+Se = the HEM diet supplemented with 0.4 mg selenium/kg from selenized yeast.

1 Feed conversion ratio is represented as feed consumption (g): egg weight (g).

2 Disqualification egg included broken eggs, salable eggs, soft shell eggs, dirty eggs, and misshapen eggs.

abc Means (n = 10) within a row with different superscripts differ significantly (P < 0.05).

### Egg quality

Dietary HEM exposure significantly decreased (*P* < 0.05) HU, albumin height, and eggshell color b^*^ and increased yolk percentage and eggshell color L^*^ compared to the CON (Table 3). Se supplementation to the HEM diet had no effect on HU and albumin height and eggshell color, but decreased yolk percentage (*P* < 0.05). However, dietary HEM exposure did not influence (*P* > 0.10) yolk color, eggshell strength, eggshell thickness, and eggshell color a^*^ (Table 3).

**Table 3:** Combined effects of dietary cadmium, lead, mercury, and chromium on selected egg quality of laying hens from 63 to 74 weeks of age and attenuated toxicity with selenized yeast.

**Items^1^**	**CON**	**Se**	**HEM**	**HEM +Se**	**SEM**	***P*** **-value**
Yolk weight, g	17.56	17.64	17.20	17.13	0.27	0.469
Yolk percentage, %	27.49^b^	28.38^b^	29.94^a^	28.42^b^	0.19	0.009
Yolk color	7.04	7.85	6.94	7.00	0.44	0.133
Haugh unit	90.26^a^	91.00^a^	84.79^b^	84.57^b^	1.52	0.019
Albumin height, mm	8.49^a^	8.55^a^	7.40^b^	6.92^b^	0.36	0.035
Eggshell thickness, mm	0.36	0.37	0.36	0.37	0.01	0.792
Eggshell strength, kg/cm^2^	3.95	3.82	3.88	3.48	0.22	0.459
Eggshell color L*^1^	82.02^a^	80.41^b^	82.33^a^	82.15^a^	0.58	<0.001
Eggshell color b*^1^	15.40^b^	17.10^a^	15.75^b^	15.56^b^	0.58	0.023
Eggshell color a*^1^	3.08	3.62	2.93	3.52	0.27	0.199

CON = the corn–soybean meal basal diet; Se = the CON diet supplemented with 0.4 mg selenium/kg from selenized yeast; HEM = the CON diet added with 5 mg cadmium/kg from CdCl_2_, 50 mg lead/kg from Pb (NO_3_)_2_, 3 mg mercury/kg from HgCl_2_, and 5 mg chromium/kg from CrCl_3_; HEM+Se = the HEM diet supplemented with 0.4 mg selenium/kg from selenized yeast.

1 L*=brightness (black/white), a*=chroma value (green/red), b^*^= chroma value (blue/yellow).

ab Means (n = 10) within a row with different superscripts differ significantly (P < 0.05).

### Accumulations of minerals in the egg

Dietary HEM exposure significantly increased (*P* < 0.05) accumulation of Cd, Pb, and Hg in the yolk compared to the CON. However, dietary HEM exposure did not influence (*P* > 0.10) accumulation of Cr and Se in the yolk. The content of Se in the HEM+Se in the yolk significantly increased (*P* < 0.05), Cd concentration significantly decreased (*P* < 0.05), but contents of Pb, Cr, and Hg had no significant difference (*P* > 0.10) compared to the HEM (Table 4). Dietary HEM exposure did not influence (*P* > 0.10) accumulation of Cd, Pb, Hg, Cr, and Se in the egg white compared to the CON. The Se supplementation did not significantly increase (*P* > 0.10) accumulation of Pb, Hg, Cd, and Cr in the egg white except Se (Table 4). The results of Pearson's correlations between accumulation of Se, Cd, Pb, Hg, and Cr in the egg yolk, egg white, and egg quality parameters are presented in Table 5. The accumulation of Cd, Pb, and Hg in the yolk was negatively correlated to egg weight, HU, and albumin height (*r* > 0.50, *P* < 0.05). Meanwhile, the accumulation of Cd in the egg white was negatively correlated to eggshell thickness (*r* > 0.50, *P* < 0.05). The accumulation of Se in the egg yolk and white was positively correlated to the b^*^ value of eggshell color (*r* > 0.50, *P* < 0.05).

**Table 4:** Accumulations of selenium, cadmium, lead, mercury, and chromium in egg yolk and egg white at 74 weeks of age of laying hens (dry matter basis).

**Items**		**CON**	**Se**	**HEM**	**HEM+Se**	**SEM**	***P*** **-value**
Cd μg/kg	Yolk	NS^1^	0.079^c^	4.41^a^	2.71^b^	0.35	<0.001
	Egg white	5.69	6.89	8.52	7.93	0.19	0.186
Pb mg/kg	Yolk	0.44^b^	0.61^b^	1.14^a^	0.99^a^	0.05	<0.001
	Egg white	0.92	0.77	1.00	1.14	0.24	0.747
Cr mg/kg	Yolk	32.92	38.15	38.30	38.64	2.69	0.405
	Egg white	24.25	22.54	23.06	25.75	1.55	0.487
Hg μg/kg	Yolk	12.49^b^	4.34^b^	235.8^a^	397.8^a^	55.99	0.001
	Egg white	9.98	4.92	8.22	7.83	1.18	0.052
Se mg/kg	Yolk	0.69^b^	1.17^a^	0.70^b^	1.33^a^	0.07	<0.001
	Egg white	0.61^b^	1.44^a^	0.64^b^	1.64^a^	0.09	<0.001

CON = the corn–soybean meal basal diet; Se = the CON diet supplemented with 0.4 mg selenium/kg from selenized yeast; HEM = the CON diet added with 5 mg cadmium/kg from CdCl_2_, 50 mg lead/kg from Pb(NO_3_)_2_, 3 mg mercury/kg from HgCl_2_, and 5 mg chromium/kg from CrCl_3_; HEM+Se = the HEM diet supplemented with 0.4 mg selenium/kg from selenized yeast.

1 NS means not detected. The limit of detection of selenium, cadmium, lead, mercury, and chromium is 0.31 μg/kg, 0.61 μg/kg, 0.87 mg/kg, 0.5 mg/kg, 0.49 mg/kg, respectively.

ab Means (n = 10) within a row with different superscripts differ significantly (P < 0.05).

**Table 5:** Correlation between accumulations of selenium, cadmium, lead, mercury, and chromium in egg and egg quality at 74 weeks of age of laying hens.

**Items**	**Egg quality parameters**	**Cd**		**Pb**		**Cr**		**Hg**		**Se**	
		***r*** **^a^**	***P*** **-value**	***r***	***P*** **-value**	***r***	***P*** **-value**	***r***	***P*** **-value**	***r***	***P*** **-value**
Egg yolk	Yolk weight, g	−0.134	0.572	−0.172	0.469	−0.010	0.935	−0.450^*^	0.047	−0.009	0.969
	Yolk percentage, %	−0.307	0.1887	−0.0617	0.798	−0.182	0.441	−0.466^*^	0.038	−0.086	0.718
	Yolk color	−0.173	0.465	−0.206	0.384	−0.331	0.154	−0.118	0.619	0.270	0.249
	Haugh unit	−0.691^**^	<0.010	−0.530^*^	0.016	−0.105	0.659	−0.684^**^	<0.010	−0.260	0.268
	Albumin height, mm	−0.659^**^	<0.010	−0.531^*^	0.016	0.130	0.584	−0.589^**^	<0.010	−0.371	0.107
	Eggshell strength, kg/cm^2^ kg/cm^2^kg/cm^2^kg/cm^2^	−0.349	0.131	−0.277	0.237	−0.105	0.659	−0.263	0.263	−0.250	0.287
	Eggshell thickness, mm	0.071	0.767	−0.043	0.856	0.258	0.273	0.180	0.447	0.185	0.436
	Eggshell color, L^*^	−0.089	0.707	−0.375	0.103	−0.310	0.183	−0.051	0.832	−0.423	0.064
	Eggshell color a^*^	0.002	0.993	0.133	0.576	0.307	0.188	0.008	0.97438	0.239	0.309
	Eggshell color b^*^	0.152	0.523	0.480^*^	0.032	0.105	0.661	0.381	0.097	0.704^**^	<0.010
Egg white	Yolk weight, g	0.019	0.942	0.288	0.218	0.053	0.825	−0.239	0.323	0.093	0.696
	Yolk percentage, %	−0.231	0.373	0.415	0.069	−0.268	0.253	−0.490^*^	0.0333	−0.069	0.772
	Yolk color	−0.108	0.681	−0.151	0.524	−0.072	0.763	−0.224	0.357	0.308	0.187
	Haugh unit	−0.333	0.192	0.072	0.764	−0.265	0.259	−0.168	0.492	−0.107	0.653
	Albumin height, mm	−0.256	0.321	−0.005	0.984	−0.239	0.311	−0.143	0.559	−0.209	0.377
	Eggshell strength, kg/cm^2^	0.188	0.470	−0.149	0.532	0.042	0.859	0.149	0.529	−0.148	0.533
	Eggshell thickness, mm	−0.552^*^	0.022	−0.149	0.532	0.042	0.859	0.107	0.662	−0.148	0.533
	Eggshell color, L^*^	−0.320	0.211	−0.181	0.446	−0.187	0.431	0.488^*^	0.034	−0.348	0.133
	Eggshell color a^*^	0.086	0.744	0.158	0.507	0.252	0.283	−0.369	0.119	0.241	0.307
	Eggshell color b^*^	0.264	0.306	0.219	0.352	0.222	0.346	−0.311	0.196	0.564^**^	<0.010

a r =correlation coefficient.

* Measurements are significantly correlated at P < 0.05;

** measurements are significantly correlated at P < 0.01 (two-tailed).

### Serum biochemical parameters

The results of serum biochemical parameters are shown in Table 6. The results showed that dietary HEM exposure significantly increased (*P* < 0.05) serum AST activity as compared to the CON, and Se supplementation significantly decreased (*P* < 0.05) the activity of AST in the serum. The dietary HEM contamination did not affect the concentrations of ALT, TP, TC, ALB, GLU, BUN, LDL-C, HDL-C, TG, E2, and P4 in the serum (*P* > 0.10) as compared to the CON, and Se supplementation did not change these indices irrespective of the CON or HEM diet.

**Table 6:** Effects of dietary selenized yeast supplementation on the serum biochemical parameters of laying hens at 74 weeks of age after feeding a cadmium, lead, mercury, and chromium contaminated diet for 12 weeks.

**Items^1^**	**CON**	**Se**	**HEM**	**HEM +Se**	**SEM**	***P*** **-value**
ALT (U/L)	3.00	2.25	2.50	2.00	0.38	0.361
AST (U/L)	177.00^c^	183.33^c^	238.67^a^	217.75^b^	7.75	0.005
TP (g/L)	50.43	51.23	50.37	49.25	3.37	0.978
ALB (g/L)	19.42	20.00	19.67	19.32	0.76	0.908
GLU (mmol/L)	13.31	13.23	12.40	11.60	0.61	0.204
BUN (mmol/L)	0.22	0.25	0.18	0.24	0.04	0.567
TC (mmol/L)	2.91	2.84	3.70	3.26	0.39	0.450
LDL-C (mmol/L)	0.88	0.98	1.14	1.23	0.15	0.385
HDL-C (mmol/L)	0.52	0.73	1.08	0.77	0.20	0.313
TG (mmol/L)	11.66	12.42	9.68	6.56	2.05	0.259
E2(pg/ml)	49.58	50.12	45.03	49.26	2.77	0.539
P4(pmol/L)	933.36	991.27	929.24	876.82	62.23	0.617

CON = the corn–soybean meal basal diet; Se = the CON diet supplemented with 0.4 mg selenium/kg from selenized yeast; HEM = the CON diet added with 5 mg cadmium/kg from CdCl_2_, 50 mg lead/kg from Pb(NO_3_)_2_, 3 mg mercury/kg from HgCl_2_, and 5 mg chromium/kg from CrCl_3_; HEM+Se = the HEM diet supplemented with 0.4 mg selenium/kg from selenized yeast.

1 ALT, glutamic-pyruvic transaminase; AST, glutamic oxalacetic transaminase; TP, total protein; ALB, albumin; GLU, glucose; BUN, serum urea nitrogen; TC, total cholesterol; LDL-C, low-density lipoprotein cholesterol; HDL-C, high-density lipoprotein cholesterol; TG, total triglycerides.

abcd Means (n = 10) within a row with different superscripts differ significantly (P < 0.05).

### Antioxidative function of different tissues

**Figure 1:**
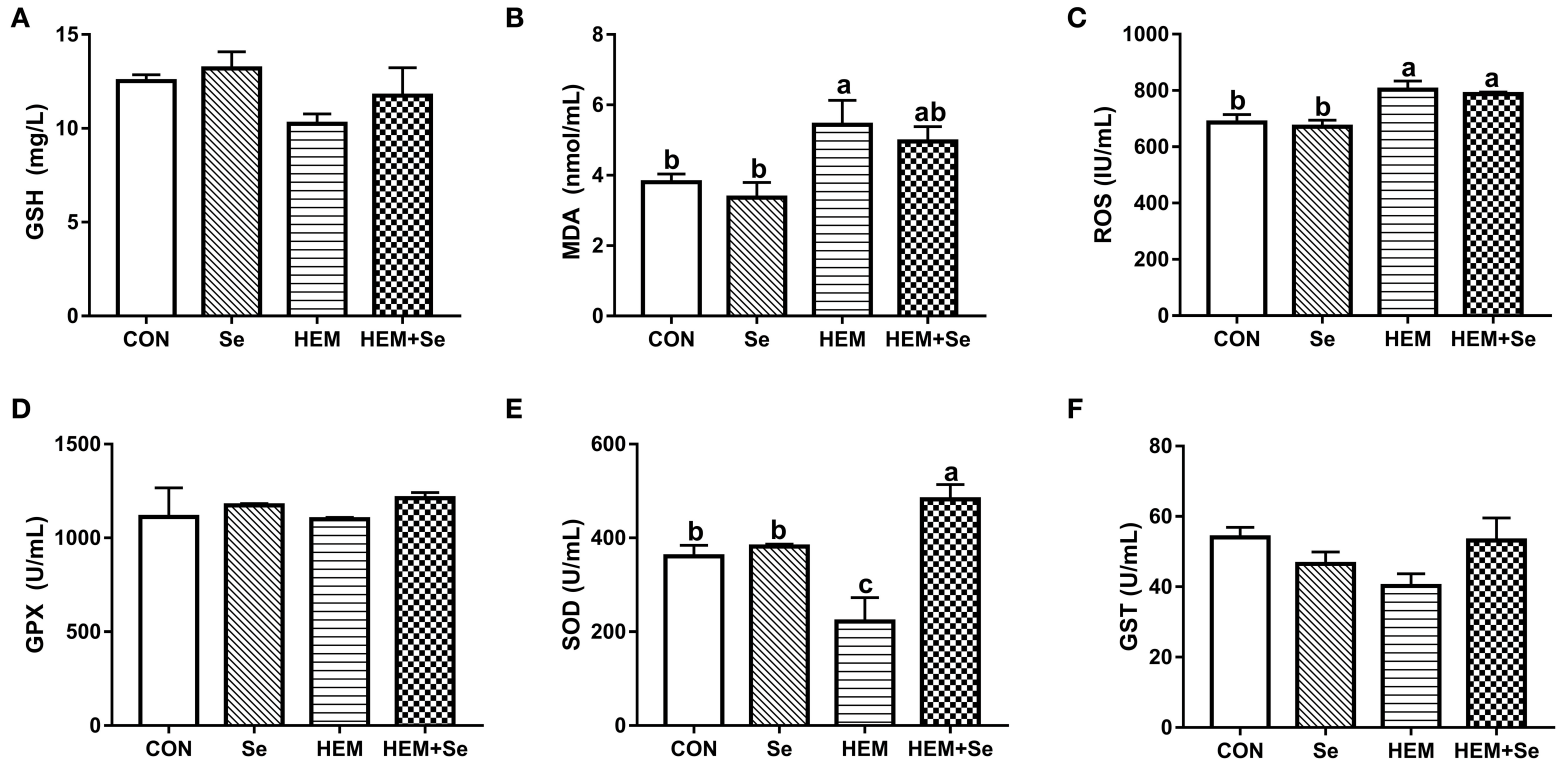
Combined effects of dietary cadmium, lead, mercury, and chromium on serum antioxidant parameters of laying hens from 63 to 74 week of age and attenuated toxicity with selenized yeast. CON = the corn–soybean meal basal diet; Se = the CON diet supplemented with 0.4 mg selenium/kg from selenized yeast; HEM = the CON diet added with 5 mg cadmium/kg from CdCl_2_, 50 mg lead/kg from Pb(NO_3_)_2_, 3 mg mercury/kg from HgCl_2_, and 5 mg chromium/kg from CrCl_3_; HEM+Se = the HEM diet supplemented with 0.4 mg selenium/kg from selenized yeast. **(B)** MDA, malondialdehyde; **(E)** T-SOD, total superoxide dismutase; **(A)** GSH, glutathione; **(D)** GSH-Px, glutathione peroxidase; **(F)** GSTs, glutathione S-transferase; **(C)** ROS, reactive oxygen species. Data are means ± SEM (*n* = 10). ^a, b^ Bars with no common superscript are significantly different (*P* < 0.05).

The results of serum antioxidative parameters are shown in Figure 1. Figure 1 illustrates that dietary HEM exposure significantly increased (*P* < 0.05) serum MDA and ROS concentrations as compared to the CON. The Se supplementation to the HEM diet had no significant effect on serum MDA and ROS concentrations (*P* > 0.10). The dietary HEM exposure significantly decreased (*P* < 0.05) serum SOD activity as compared to the CON. The Se supplementation significantly increased (*P* < 0.05) serum SOD activity for the HEM diet, but not for the CON diet. The dietary HEM exposure had a tendency (*P* = 0.09) to decrease serum GST activity as compared to the CON. The HEM-Se treatment tended to increase serum GST activity as compared to the HEM treatment (*P* = 0.09). However, dietary HEM exposure or Se supplementation did not affect (*P* > 0.10) serum GPX and GSH activities as compared to the CON.

**Figure 2:**
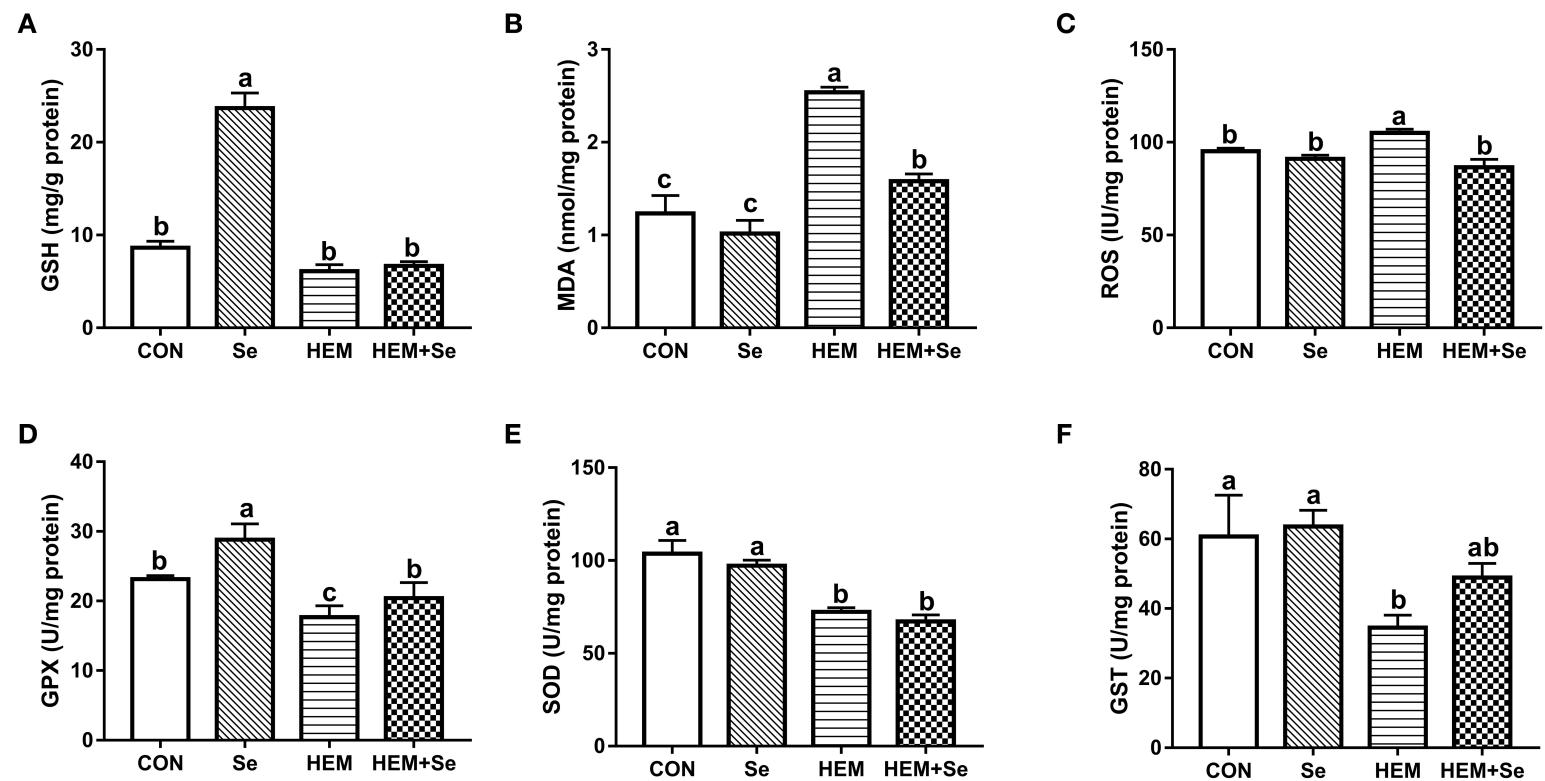
Combined effects of dietary cadmium, lead, mercury, and chromium on liver antioxidant parameters of laying hens from 63 to 74 weeks of age and attenuated toxicity with selenized yeast. CON = the corn–soybean meal basal diet; Se = the CON diet supplemented with 0.4 mg selenium/kg from selenized yeast; HEM = the CON diet added with 5 mg cadmium/kg from CdCl_2_, 50 mg lead/kg from Pb(NO_3_)_2_, 3 mg mercury/kg from HgCl_2_, and 5 mg chromium/kg from CrCl_3_; HEM+Se = the HEM diet supplemented with 0.4 mg selenium/kg from selenized yeast. **(B)** MDA, malondialdehyde; **(E)** T-SOD, total superoxide dismutase; **(A)** GSH, glutathione; **(D)** GSH-Px, glutathione peroxidase; **(F)** GSTs, glutathione S-transferase; **(C)** ROS, reactive oxygen species. Data are means ± SEM (*n* = 10). ^a, b, c^ Bars with no common superscript are significantly different (*P* < 0.05).

The results of antioxidative parameters in the liver are shown in Figure 2. From Figure 2, it can be seen that dietary HEM exposure significantly increased (*P* < 0.05) liver MDA and ROS concentrations as compared to the CON. The Se supplementation significantly decreased (*P* < 0.05) liver MDA and ROS concentrations for the HEM diet, but not for the CON diet; however, liver MDA concentration was higher in HEM-Se group than that in the CON or Se groups. The HEM treatment significantly decreased (*P* < 0.05) liver SOD, GST, GPX, and GSH activities as compared to the CON group. The Se supplementation significantly increased (*P* < 0.05) liver GPX and GSH activities for the CON diet, but it only increased liver GPX activity for the HEM diet.

**Figure 3:**
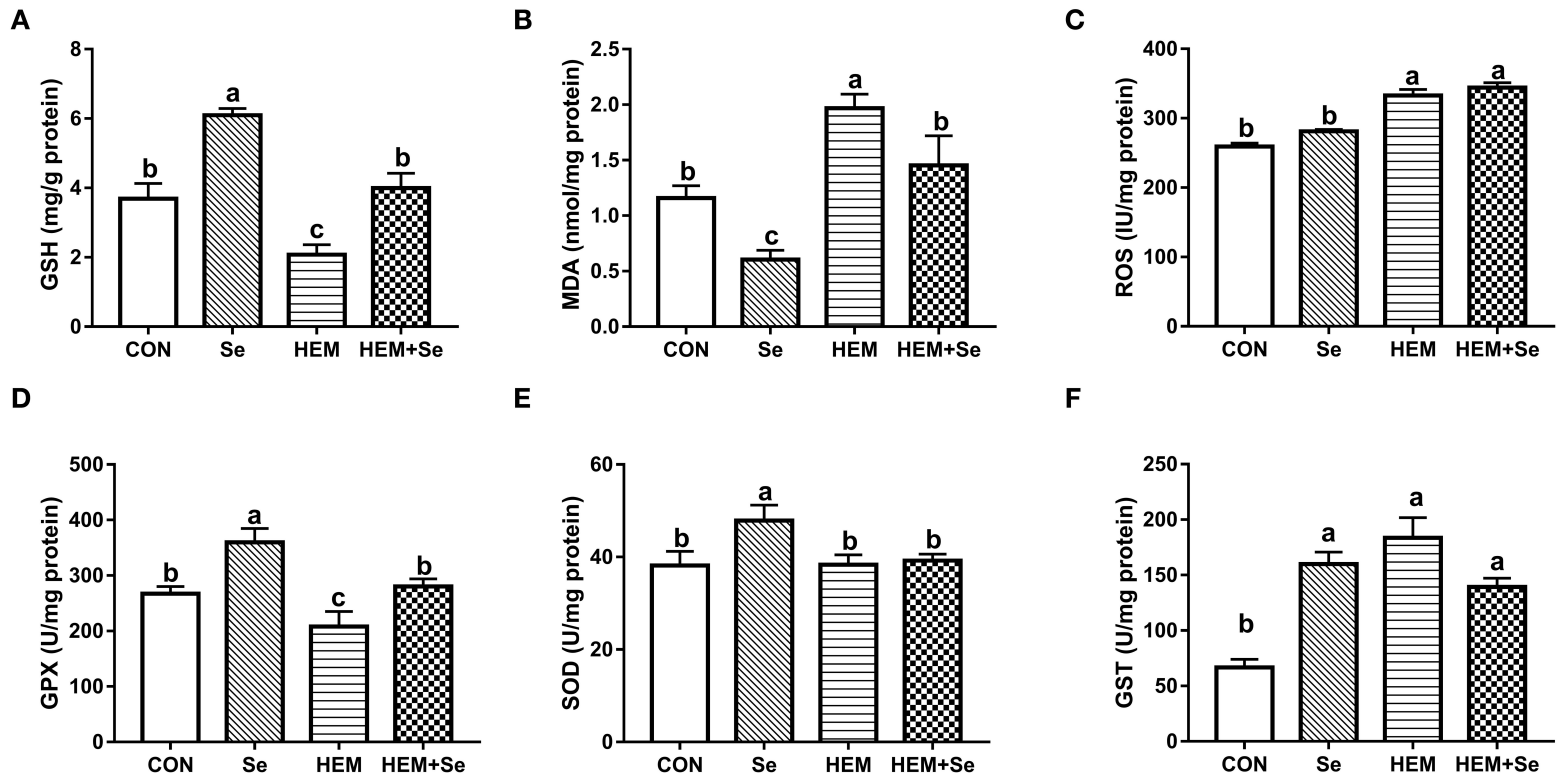
Combined effects of dietary cadmium, lead, mercury, and chromium on ovary antioxidant parameters of laying hens from 63 to 74 weeks of age and attenuated toxicity with selenized yeast. CON = the corn–soybean meal basal diet; Se = the CON diet supplemented with 0.4 mg selenium/kg from selenized yeast; HEM = the CON diet added with 5 mg cadmium/kg from CdCl_2_, 50 mg lead/kg from Pb(NO_3_)_2_, 3 mg mercury/kg from HgCl_2_, and 5 mg chromium/kg from CrCl_3_; HEM+Se = the HEM diet supplemented with 0.4 mg selenium/kg from selenized yeast. **(B)** MDA, malondialdehyde; **(E)** T-SOD, total superoxide dismutase; **(A)** GSH, glutathione; **(D)** GSH-Px, glutathione peroxidase; **(F)** GSTs, glutathione S-transferase; **(C)** ROS, reactive oxygen species. Data are means ± SEM (*n* = 10). ^a, b, c^ Bars with no common superscript are significantly different (*P* < 0.05).

The results of antioxidative parameters in the ovary are shown in Figure 3. Figure 3 illustrates that dietary HEM exposure significantly increased (*P* < 0.05) ovary MDA and ROS concentrations as compared to the CON. The Se supplementation significantly decreased (*P* < 0.05) ovary MDA concentration when compared to the HEM treatment. The dietary HEM exposure significantly decreased (*P* < 0.05) ovary GPX and GSH activities, but increased GST activity as compared to the CON treatment. The Se supplementation increased ovary GPX and GSH activities irrespective of for the CON or HEM diet, but it only increased ovary SOD activity for the CON diet. However, dietary HEM exposure did not influence ovary SOD activity as compared to the CON.

## Discussion

Cadmium, lead, mercury, and chromium are common environmental pollutants and frequently are found together, and their concentrations are high in manure and feedstuff (39–41). Their permitted limits are regulated by the Chinese Hygienical Standard for Feeds. To construct the laying hen model of sub-chronic heavy metal intoxication, the addition of 5 mg/kg Cd, 50 mg/kg Pb, 3 mg/kg Hg, and 5 mg/kg Cr with their common chemical structures [CdCl_2_, Pb (NO_3_)_2_, HgCl_2_, and CrCl_3_] in manure and feedstuff, approximate the 10-fold of the limits of national standards, was chosen in this study, and the test period was 12 weeks. Their contents in basal diet were Cd 0.18 mg kg^−1^, Pb 2.11 mg kg^−1^, Hg 0.01 mg kg^−1^, and Cr 7.68 mg kg^−1^, respectively.

In this study, our results suggested that dietary HEM exposure decreased laying performance and egg white quality (HU and albumin height) of laying hens during a 12-week trial period. It has been reported that Cd, Pb, Hg, and Cr alone or combination have negative effects on laying performance or egg quality of female poultry. Cd at 15–150 mg/kg feed as CdCl_2_ or CdSO_4_, Pb at 1–60 mg/kg as Pb (C_2_H_3_O_2_)_2_ or Pb (NO3)_2_, Hg at 3.325 mg/kg as HgCl_2_, and Cr as Cr propionate at 400 μg/kg to some extent reduced laying performance and egg quality in poultry (8, 9, 12, 14, 15, 17). Furthermore, combination of Pb and Cd, or Pb and Hg, or Pb, Hg, and Cd decreased laying performance and egg quality and impaired hepatic function and reproductive system function of female poultry (26–30). Our findings were supported and strengthened by the above research. Combining the above antioxidative results, we speculate that high uptake of heavy metals and multiple HEM reduces laying performance and egg quality of laying hens by increasing stress sensitivity and disrupting hepatic dysfunction. Meanwhile, two or more than two kinds of heavy metals with different sources may have synergistic toxic effect on oxidative status, performance, and egg quality (26). Anyone of HEM may be the one that predominantly affected oxidative status or laying performance due to its more reactive nature (46). HEM had accumulation characters. The result of herein suggested that HEM exposure time may contribute to HEM toxicity to laying performance and egg quality of laying hens.

It has been reported that heavy metals are a powerful inducer of oxidative stress, and oxidative stress is considered to be a primary reason for heavy metal-induced hepatic, renal, and reproductive toxicity. In the present study, dietary HEM exposure induced higher MDA and ROS in the serum, liver, and ovary and decreased the activity of SOD in the serum, the activity of SOD, GST, GPX, and GSH in the liver, and the activity of GPX and GSH in the ovary. These results indicate that dietary HEM exposure resulted in oxidative stress to laying hens. Dietary HEM exposure significantly increased (*P* < 0.05) AST concentrations in the serum compared to the CON indicating that HEM caused hepatic dysfunction.

One of the important mechanisms underlying their toxicity is oxidative stress which induces reactive oxygen species and/or depletes the antioxidant defense system (47). The Cd, Pb, Cr, and Hg absorbed from the digestive system are transported to the bloodstream by binding to metallothionein or others, such as globulin, albumin, cysteine, and glutathione (GSH), or sulfhydryl (-SH) groups, such as superoxide dismutase (SOD), catalase (CAT), and glutathione peroxidase (GPx), and then transported to tissues, such as liver, kidneys, and reproductive organs. When the amount of HEM in the circulatory system is greater than the binding capacity of different types of protein or sulfhydryl (-SH) groups, the free HEM induces ROS and MDA, affects many enzymes and other SH-containing molecules, and depletes the antioxidant defense system, finally, resulting in oxidative damage to the liver and reproductive organs.

However, some contrasting results were observed. Hg at 25–100 mg/kg as ethyl Hg chloride, and combination of Pb (30 mg/kg) and Hg (1.2 mg/kg) did not significantly depress egg quality of laying hens (16, 28). Some previous studies have shown that organic or inorganic Cr had no effect or a slight increase in egg production (20–22). All in all, Cd, Pb, Hg, and Cr toxicity varies considerably depending on the time of exposure, species, gender, the amount, and duration of intake and environmental and nutritional factors (48).

In our present study, Cd and Pb concentration in the egg yolk was lower than those in the egg white which may contribute to HEM depress egg white quality. Hg and Cr concentration in the egg white was lower than those in the egg yolk. However, the results of Pearson's correlation analysis suggest the accumulation of Cd, Pb, and Hg in the yolk was negatively correlated to HU, and albumin height (*r* > 0.50, *P* < 0.05). The abovementioned results imply that HEM depressed egg white quality related to the HEM accumulation in the egg white or the egg yolk. Yan, Ma et al. (49) found that both albumen height and HU were negatively correlated to the accumulation of Hg in albumen and yolk, and Hg concentration was transferred from yolk to albumen with Hg dosage increasing in diet. Our data were consistent with this report. The possible reasons were that inorganic Hg bound with protein in the ovary or oviduct of laying hens is converted to organic Hg and deposited in yolk and albumen which ultimately affected egg white quality.

Hen-day egg production of laying hens depends on follicle sequencing. E2 and P4 are secreted by the ovary and play an important role in follicle development (50). Nolan and Brown (51) found that 27 mg Cd/kg as CdCl_2_ decreased serum E2 of laying hens. Georgescu et al. (52) reported that Pb might exhibit endocrine-disrupting activity in animals, acting as a powerful disruptor of adrenal and ovarian steroidogenesis, inhibiting the synthesis and activity of progesterone. Yuan et al. (15) found that 15 to 60 mg/kg of Pb depressed progesterone. The possible reasons were that Pb might bind to the steroid hormone receptors after accumulation in the ovary and suppress E2 and progesterone secretion. Yan et al. (49) reported that 3–27 mg/kg of Hg as HgCl_2_ decreased progesterone and increased atretic follicle number. Our results indicate that dietary HEM exposure did not significantly affect serum E2 and P4 of laying hens. We speculate the possible reasons were that dosages of HEM were too lower to disrupt E2 or P4. In addition, another reason may be that any one element has antagonistic effect with another or others resulting in HEM depressing toxicity on hormone.

Selenium is an essential dietary trace element. Generally speaking, inorganic Se and organic Se are two major Se sources for poultry. Inorganic Se mainly includes selenite or selenate. Due to higher toxicity, interactions with other, minerals, and vitamins, low efficiency of transfer to milk, meat, and eggs of inorganic Se, and their use were limited in commercial poultry (53). It was well known that organic Se was effective resource of Se in poultry and animal production which is related to its ability to build Se reserves in the body and higher bioavailability. Organic Se mainly was selenized yeast in the form of selenomethionine (SeMet). Selenized yeast used in this study contains about 60% SeMet which was similar with the content presented in literature (54). It was confirmed that SeMet was the major form of Se in grains, oil seeds, and other important feed ingredients (55). Therefore, we speculate that the Se in the basal diet was SeMet too.

It has been shown that SeMet can increase antioxidative function of animals against environmental stresses (56). Our data suggested that Se as selenized yeast showed an ameliorative effect on oxidative damage in the serum, liver, and ovary by decreasing MDA or ROS and partly decreasing the activity of antioxidant enzymes. Previous reports have revealed that adequate doses of organic or inorganic Se can eliminate hydrogen peroxide (H_2_O_2_), lipid, and phospholipid hydroperoxides and protect tissues such as liver, kidneys, brain, and reproductive organs from individual Cd, Pb, Cr, and Hg-induced or combined Pb and Cd-induced oxidative damage (32–35, 38), which are consistent with the work reported herein. Other possible reasons were that SeMet inhibits HEM-induced stress. Second, Se could exert an antidotal action through the formation of insoluble complexes consisting of HEM selenide (HEMSe) through the binding mechanism (57). Third, Se is a component of selenoprotein, which exhibits a critical role during the biological processes. SeMet can be available for selenoprotein synthesis, be released from tissues, and be efficient in maintaining the GPX level (53, 54). Newairy et al. (58) also pointed out that the protective potential of Se may be associated with the recovery of GPX and SOD activities. We also found that Se improved SOD activity in the serum, GPX activity in the liver and GPX activity, and GSH concentration in the ovary. The toxicity of HEM on oxidative stress and the ameliorative effect of Se are different in different tissues in the study. It has been reported that the toxicity of HEM was related to the cell type of tissues. Some somatic cells in the target tissue of HEM have a high affinity, while others have low affinity (32, 56). The different accumulation of free HEM and Se may also account for the question.

Our data suggested that Se addition of 0.4 mg/kg based on the basal diet of 1.4 mg/kg decreased AST concentrations in the serum. In other words, Se addition ameliorated hepatic dysfunction. The possible reason was that Se modulated hepatic inflammation-related genes and element homeostasis and attenuated oxidative damage of the liver (32, 59).

The data reported herein demonstrate that Se retention in the egg white was more than that in the egg yolk, and Se addition did not elevate Cd, Cr, Pb, and Hg content in the egg yolk and egg white. Swanson and Christine (60) found selenized yeast promotes deposition into egg white. Our results are consistent with this report. Stress or disease conditions make laying hens lay lighter-colored eggshells (61). In this study, the accumulation of Se in egg yolk and white was positively correlated to the b^*^ value of eggshell color. L^*^a^*^b^*^= L^*^ – a^*^ – b^*^, lower is the value (L^*^a^*^b^*^), dark is the egg. These indicate that Se contributes to improving egg quality through its antioxidant ability. However, selenized yeast in this study did not alleviate the HEM toxicity on egg white quality which may be associated with Se addition dosage. Although the permitted limit of Se is 0.5 mg/kg diet in China (42), it has been shown that selenosis can occur in laboratory animals, livestock, and humans following long-term exposure to 5 mg Se/kg of diet (62). The National Academy of Sciences has accepted 5 mg Se/kg diet as the division level between toxic and non-toxic feeds (63). Thus, we assume that Se addition of 0.4 mg/kg cannot fully offset the toxicity of HEM on egg white quality. Se dosages should vary for nutrition, disease-resistant, and enrichment purpose which should be explored in future.

Given the co-exposure to multiple heavy metals is a more realistic scenario, the effects of these metals on oxidative status when simultaneously present in the organism have become one of the contemporary issues in toxicology. The combined effect of multiple heavy metals and their additive, synergistic, antagonistic, or independent effects, and their molecular mechanisms needs further investigation.

## Conclusion

In conclusion, dietary HEM exposure depressed laying performance and egg white quality which are likely associated with HEM impaired antioxidant capacity, disrupted hepatic function, and elevated HEM accumulation in the egg yolk and egg white. Selenium as selenized yeast addition of 0.4 mg/kg attenuated toxicity of HEM on laying performance, oxidative stress, and hepatic function, but did not ameliorate the effects of HEM toxicity on egg white quality. The interactive relationship of HEM and exact selenium dosages for disease-resistant purpose perhaps need to be explored in future.

## Data availability statement

The original contributions presented in the study are included in the article/supplementary material, further inquiries can be directed to the corresponding author.

## Ethics statement

The animal study was reviewed and approved by Sichuan Agricultural University Animal Ethical and Welfare Committee.

## Author contributions

CW, SB, and KZ: conceptualization. LL, GL, SB, JW, and YL: methodology. LL, YJ, HL, FW, and BW: investigation. SB, CW, JS, and LL: data curation and writing—original draft preparation. CW, SB, LL, BW, HL, FW, and YJ: formal analysis. GL, YL, JW, and TA: supervision. SB and CW: funding acquisition. All authors reviewed, edited, and approved the final version of the manuscript.

## Funding

This work was supported by the National Key Research and Development Program of China (No. 2021YFD1300203).

## Conflict of interest

Author BW was employed by Chelota Biotechnology Co., Ltd. The remaining authors declare that the research was conducted in the absence of any commercial or financial relationships that could be construed as a potential conflict of interest.

## Publisher's note

All claims expressed in this article are solely those of the authors and do not necessarily represent those of their affiliated organizations, or those of the publisher, the editors and the reviewers. Any product that may be evaluated in this article, or claim that may be made by its manufacturer, is not guaranteed or endorsed by the publisher.
